# Backward Walking Styles and Impact on Spatiotemporal Gait Characteristics

**DOI:** 10.3390/healthcare10122487

**Published:** 2022-12-09

**Authors:** Teerapapa Luecha, Shin Takesue, Wen Liang Yeoh, Ping Yeap Loh, Satoshi Muraki

**Affiliations:** 1 Department of Human Sciences, Graduate School of Design, Kyushu University, Fukuoka 815-8540, Japan; 2 Faculty of Sciences and Engineering, Saga University, Saga 840-8502, Japan; 3 Department of Life Design and Science, Faculty of Design, Kyushu University, Fukuoka 815-8540, Japan

**Keywords:** backward walking, spatiotemporal gait, center of mass, visual feedback, arm position, speed

## Abstract

Forward walking (FW) is a common balance assessment tool. However, its sensitivity is limited by the ceiling effect. Reverse gait, such as backward walking (BW), has been reported to have more advantages than FW for balance assessment. Three factors related to postural instability (i.e., increased speeds, restricted arm swing, and reduced visual feedback) during BW were investigated to determine BW conditions that have the potential to predict falls. Three-dimensional analyses were used to analyze seven walking conditions. FW and BW at self-selected and fast speeds were analyzed to identify the effects of speed. Walking with normal arm swings, crossed arms, and abducted arms during BW was tested to determine the effects of arm position. BW with closed and open eyes was compared to investigate the effects of visual feedback. BW had a significantly shorter step length than FW at high speeds. When the arms were abducted, the stance phase (%) was significantly lower compared to when arms were crossed during BW. Moreover, BW with closed eyes revealed significantly higher mediolateral center of mass (COM) displacements than with open eyes. We observed that BW with fast speeds, a crossed arm position, and closed eyes has the potential to help assess fall risk because it requires higher balance ability through spatiotemporal and COM adjustment.

## 1. Introduction

Falls are globally the second most common cause of death due to unintentional injuries [1]. They are often foreseeable; therefore, they can be prevented. The four predictive factors with the strongest association with falls include (1) impairment of balance, gait instability, and dizziness; (2) impairment of memory and judgment; (3) impairment of body movement function by decreased muscle power; and (4) experienced falls [2]. Fall indicators are provided by balance assessments and daily movement evaluation tools (e.g., questionnaire, sit-to-stand, standing, reaching, posture transition, gait, etc.) to screen for the risk of falls.

In addition to balance assessment tools, gait analysis is a powerful means by which clinicians may detect the risk of falls and diagnose pathologies. Pathologies and aging can lead to the deterioration of multiple neurological structures that decrease gait performance, which can be observed through abnormalities in movement, such as excessive sway, missteps during walking, and momentum imbalance during posture transitions [3]. Biomechanical analyses of gait changes at slower speeds, shorter step lengths, wider step widths, longer stance phases, and greater center of mass (COM) displacements are often used to assess the risk of falls; however, COM displacements may result in gait changes that are unobservable by human vision [4].

A large proportion of falls occur during ambulation. Thus, most gait balance tests measure gait speed (e.g., timed up and go test, 10 m walk test) during a simple daily task such as forward walking (FW) [5]. However, there is strong evidence that these gait balance tests are limited by the ceiling effect [6] and are not sufficiently sensitive to measure balance in healthy elderly [7,8] and young adults [8]. A previous study used backward walking (BW) in the timed up and go test and demonstrated that BW is more closely related to an individual’s balance and history of falls compared to forward walking. It concluded that the use of BW improves the precision of predicting falls among healthy elderly individuals [9], and slower BW gait is correlated with a higher risk of falls in the elderly, which is also supported by the results of similar studies [10]. Moreover, some researchers have suggested that increasing speeds during BW can be used to assess dynamic balance and mobility deficits in independent community-dwelling older adults [11].

BW appears to be a valuable tool for increasing sensitivity in the gait balance test, as it is more challenging to maintain postural stability when the visual inputs for forthcoming events are decreased [12]. It improves training and rehabilitation compared to FW. BW training is recommended to improve functional balance [13] and gait mobility [14]. Biomechanical research indicates that BW is a timed reverse gait pattern with a lower velocity, step length, and stride time but an increased stance phase than FW [15,16].

Despite numerous studies on the advantages of BW, there is no strong evidence that BW can predict fall risk. Gait performance is a fall indicator, but performance parameters are rarely reported, especially for BW. Gait requires the use of higher-level cognitive, attention, and executive functions to incorporate multiple sensory feedbacks (e.g., visual, somatosensory, auditory, etc.), enabling efficient energy expenditures and the maintenance of stable forward progression of COM [17,18]. Dynamic postural stability is maintained by controlled COM displacements that retain the COM within the base of support (BOS), usually achieved by the coordination of interlimb, trunk, and pelvic twist movements [17]. Moreover, trunk and pelvic twist coordination during voluntary movements increase the amplitudes of the upper and lower limbs during increases in gait speed for maintaining postural stability [19]. According to Bovonsunthonchai et al. [20], coordination of the upper and contralateral lower limbs is necessary to improve gait performance in stroke-injured individuals.

The effects of gait speed, arm positions, and visual feedback on BW are not fully understood. Thus, this study aims to investigate the effects of these three parameters on BW in young adults. BW was performed with increasing speed, decreasing arm swing, and no visual feedback. First, a higher walking speed was hypothesized to affect gait parameters more closely related to fall risk during BW than FW. Second, enforcing a walking posture or removing visual feedback during BW was hypothesized to increase the challenge of the BW task and improve sensitivity to impairments in balance or postural control in young adults.

## 2. Materials and Methods

### 2.1. Participants

Twenty-three healthy young adults participated in this study. None of the participants had injuries or experienced lower extremity pain in the last 6 months. Participants were able to walk independently without assistive devices. This study was approved by the Research Ethics Committee, Faculty of Design, Kyushu University. Informed consent was obtained before the study procedure and the experiments.

### 2.2. Experimental Conditions of Walking

This study considers seven walking conditions performed for a 5 m distance in the forward and backward directions while looking straight ahead. The instructions given differed depending on the factors analyzed in this study.

First, we investigated the effects of walking direction and speed by comparing FW and BW at two walking speeds. FW was considered the baseline condition. The participants were instructed to walk at a self-selected speed and as fast as safely possible for both FW and BW. Normal forward walking (NFW) was defined as the self-selected speed walking in the forward direction, and fast forward walking (FFW) was defined as forward walking with the fastest speed while walking safely. Likewise, normal backward walking (NBW) refers to BW at a self-selected speed, and fast backward walking (FBW) refers to BW at the fastest safe speed.

**Figure 1:**
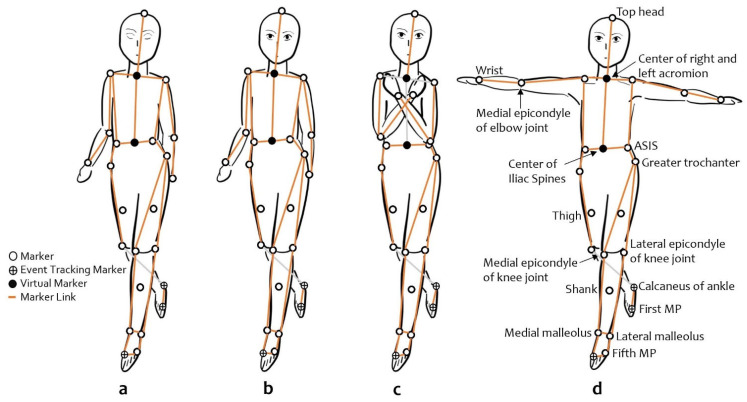
Backward walking conditions at self-selected speeds show a subset of the markers used for gait analysis: (**a**) closed eyes backward walking; (**b**) normal backward walking, the control condition used to analyze the effect of arm swing and visual feedback, where participants walked independently at a comfortable speed with arm movements; (**c**) crossed arms backward walking condition; (**d**) abducted arms backward walking condition is the posture similar to a T-pose. MP—metatarsophalangeal joint; ASIS—anterior superior iliac spine.

Second, we investigated the effects of arm position. Three different arm positions during BW at self-selected speeds were compared (Figure 1). Arm positions were designed to restrict arm swing. NBW (with normal arm swing) was the control condition and was compared to crossed arms during backward walking (CrossBW) and abducted arms during backward walking (AbBW). CrossBW was defined as both arms crossed over the chest with hands placed on opposite shoulders during self-selected BW, while AbBW was defined as 80–90° bilateral shoulder abduction during self-selected BW.

Third, we investigated the effects of visual feedback and compared two self-selected BW with different visual conditions. NBW (independent BW with eyes open) was the control condition that was compared to closed eyes backward walking (ClosBW), which had no visual feedback. ClosBW was defined as the self-closing of both eyes during a self-selected speed BW (Figure 1).

### 2.3. Experimental Procedure

Participants were asked not to perform any strenuous activity within 24 h prior to the experiment. The participants walked barefoot and wore tight-fitting clothes and caps during the experiment. Before taking any measurements, the participants performed practice trials to ensure they were able to follow the instructions and perform the tasks safely. The order of the seven walking conditions in this study was randomized for each participant. Three successful trials were performed for each condition.

### 2.4. Measurement

A three-dimensional motion analysis using Cortex™ motion analysis software, consisting of 10 infrared cameras (Motion Analysis Corporation, Santa Rosa, CA, USA) and 9.5 mm diameter reflective markers, was used to record the participants’ motion at 100 Hz. The motion data were filtered using a 6 Hz Butterworth low-pass filter. Thirty-five reflective markers were attached to the bony landmarks of the participants’ bodies as follows: head (fore, upper, rear), upper limbs (acromion of shoulders, medial epicondyle of elbows, ulnar styloid process of wrists), vertebral (7th cervical) and pelvic regions (anterior and posterior superior iliac spine), and lower limbs (greater trochanter, thighs [center point between hip joint and knee joint], lateral and medial epicondyle of femurs, shanks [center point between knee joint and ankle joint], lateral and medial malleolus, calcaneus of the ankle, and 1st and 5th metatarsophalangeal joints). In addition, one gait cycle was measured in each trial. The average of three complete trials for each walking condition was analyzed using KineAnalyzer software (Kissei Comtec, Nagano, Japan).

Velocity (m/s) was calculated as the total distance divided by the total time of the gait cycles, and cadence (steps/min) was calculated from the time taken to complete one gait cycle. The step lengths (mm) were calculated as the distance between the initial contact point of one foot and the initial contact point of the contralateral foot, and the mean of the step lengths was presented in this study. The mean step length divided by cadence was represented as the walk ratio (mm/steps/min). Finally, the step width (mm) was evaluated as the distance between the lines through the midline of the two consecutive step heels during a double stance.

**Figure 2:**
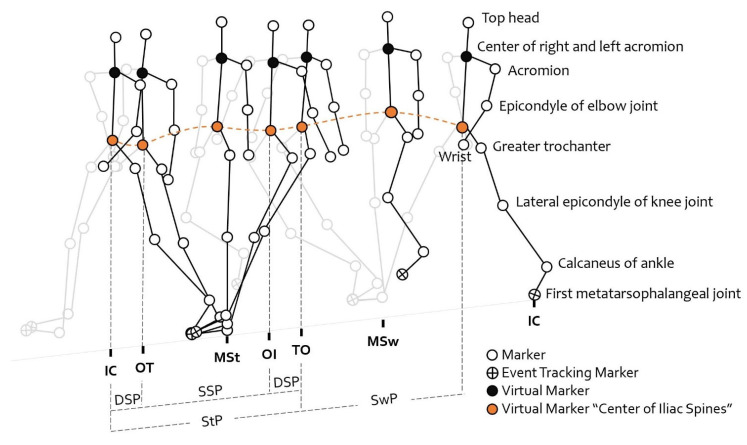
Backward gait cycle. The midpoint of the iliac spine was plotted as a representative marker for all events. The events were marked as initial contact (IC), opposite toe-off (OT), opposite initial contact (OI), toe-off (TO), and the subsequent IC. The phases of the gait cycle were estimated using mid-iliac-spine plots. Double support phase (DSP) is the period from IC to OT and the period from OI to TO, and single support phase (SSP) is the period from OT to OI. Stance phase (StP) refers to the total of DSP and SSP, while swing phase (SwP) refers to the period from TO to IC.

The gait cycle (Figure 2) was divided into the stance phase (StP) and swing phase (SwP). StP was further subdivided into the single stance phase (SSP) and double support phase (DSP). These phases were calculated using the time stamps of the following gait events: initial contact (IC), opposite toe-off (OT), opposite initial contact (OI), and toe-off (TO). These events were identified using the motion of the first metatarsophalangeal joint and the ankle calcaneus markers. Specifically, the gait period was calculated as the time taken between IC and the subsequent IC of the same foot. StP was calculated as the duration between IC and TO, while SwP was calculated as the duration between TO and IC. DSP was calculated by summing the periods between IC and OT and between IO and TO. StP, SwP, and DSP were also calculated as percentages (%) of the gait cycle by dividing their duration by the stride time.

Additionally, the total body COM was evaluated using KineAnalyzer, which uses a simplified seven-segment model described by Ebara and Yamamoto [21], which consists of the trunk, two upper legs, two lower legs, and two feet. The COM of the trunk segment was estimated as 66% of the body mass at 65% of the distance from the center of the left and right greater trochanters to the center of the right and left acromions. The COM of the upper-leg segments was estimated as 10% of the body mass located at 55% of the distance from the lateral epicondyle of the knee to the greater trochanter, while the COM of two lower legs was estimated as 5% of the body mass located at 55% of the distance from the lateral malleolus to the lateral epicondyle of the knee. The COM of the feet segments was estimated as 2% of the body mass located at 50% of the distance from the fifth metatarsal bone to the lateral malleolus. The vertical COM displacement (VT COM) and mediolateral COM displacement (ML COM) refer to the range of motion of the COM in the vertical and mediolateral directions, respectively.

### 2.5. Statistical Analysis

Statistical analysis was performed using IBM SPSS Version 23.0 (Chicago, IL, USA). Descriptive results are presented as means and standard deviations (mean ± SD). The main and interaction effects of gait speed and direction were analyzed using two-way repeated measures ANOVA. The interaction effect findings were analyzed using the Bonferroni test. The differences in gait parameters among the BW arm positions were analyzed using one-way ANOVA. The paired *t*-test was used to determine the visual feedback effect during BW. The significance level was set as α = 0.05.

## 3. Results

The data from 23 participants were included in the analysis. The participants’ characteristics are listed in Table 1.

### 3.1. The Effect of Walking Direction and Speed

As shown in Table 2, a two-way repeated measures ANOVA was performed. The effect of speed demonstrates a significant difference between the self-selected speed and fast speed in different gait directions on spatiotemporal parameters, phases of the gait cycle, and VT COM. The effect of direction shows a significant difference between FW and BW at different speeds on velocity, step length, step width, walk ratio, phases of the gait cycle, VT COM, and ML COM. In addition, the interaction effect (direction × speed) shows a significant difference in cadence, step length, stance phase, and swing phase.

Subsequently, a post hoc test revealed a significant increase in cadence (*p* < 0.001, *p* < 0.001), step length (*p* < 0.001, *p* < 0.001), and stance phase (*p* < 0.001, *p* < 0.001) but a decrease in swing phase (*p* < 0.001, *p* < 0.001) during increased walking speeds compared to the self-selected speeds during FW and BW, respectively. The NBW group showed a significantly lower cadence (*p* = 0.011), step length (*p* < 0.001), and swing phase (*p* = 0.005) and a longer stance phase (*p* = 0.008) than the NFW group. FBW also demonstrated a shorter step length (*p* < 0.001) than FFW at increased speeds.

### 3.2. The Effect of Arm Position

Regarding the results presented in Table 3, there are significant differences in the stance phase (%), swing phase (%), and DSP (%) among these conditions. A post hoc Bonferroni test showed a significantly longer stance phase (%) (*p* = 0.034) and DSP (%) (*p* = 0.019) and a shorter swing phase (%) (*p* = 0.034) during CrossBW than during AbBW. However, there are no significant differences in the spatiotemporal and COM parameters of BW at different arm positions.

### 3.3. The Effect of Visual Feedback on Backward Walking

The comparison between open and closed eyes during BW was analyzed using a paired *t*-test as presented in Table 4. The results demonstrate greater ML COM during ClosBW than during NBW. In contrast, no significant differences were found in the spatiotemporal parameters of ClosBW and NBW.

## 4. Discussion

The results demonstrate that speed, arm position, and visual feedback affect BW in young adults. The analysis of the various BW conditions in this study reveals that these three factors affect spatiotemporal parameters and COM displacement during BW. The results for the three factors are discussed in separate sections.

### 4.1. The Effect of Direction and Speed on Backward and Forward Walking

As expected, cadence and step length increased significantly as walking speed increased, whether walking forward or backward. Step length and VT COM displacements also increased significantly with increasing walking speed, consistent with the results of numerous studies [22,23,24]. Furthermore, in line with the results reported by Laufer [16], we found that the BW conditions have a significantly lower gait velocity, shorter step lengths, wider step widths, and a lower walk ratio than FW, regardless of speed. Additionally, previous studies have reported a slower velocity and shorter step length in self-selected gait speeds during BW compared to FW [15,25,26]. In agreement with Bogen et al. [27], walk ratios at self-selected speeds during BW were significantly lower than those during FW, indicating lower gait control during BW at all speeds.

Several studies have demonstrated that hip, knee, and ankle joint movements during BW are almost similar to those during FW in the time-reversed pattern [15,25,28]. A study of joint kinematics reported that a few points of peak hip and knee joint movement during FW are not performed during BW, and the knee joint during BW generates significantly lower maximum shock absorption than for FW in the loading response phase [15]. From the loading response to the next initial contact during BW gait period, the degree of hip joint flexion and extension was also lower than during FW [15,25]. Likewise, muscle activation of the hip extensors, such as the gluteus maximus, was observed to be of a lower magnitude or often almost absent during BW compared to FW [28]. The decrease in hip joint movement could have contributed to the decreased step lengths, which led to a slower velocity during BW. Accordingly, to compensate for the lower hip movement during BW, a person might adjust their walking strategy by using the hip abductors or adductors to maintain balance by increasing the step width. Compared to FW, a person may not be able to walk with the same step length and width during BW regardless of speed. All these hint that BW gait should be analyzed separately from FW gait as they result from very different body strategies.

In our results, the ML COM displacement during BW was significantly greater than that during FW regardless of speed. Nonetheless, previous studies on COM displacement during BW are limited. A higher level of cognitive function can decrease errors or internal perturbations in FW gait by using visual cues, such as walkways, obstacles, objects, and environments, to predict forthcoming events [29]. Because forthcoming events are unpredictable during BW, internal perturbations may be induced and lead to more significant ML COM displacements due to decreased sensorimotor control signals, reduced visual cues, and unusual tactile signals. Essentially, COM movements must be controlled within the BOS to maintain balance [30].

The COM–BOS relationship involves the generation of internal forces to maintain postural stability. Biomechanically, voluntary movement compensates using proactive postural adjustments to maintain postural control during COM perturbations. Thus, walking steps are widened to sustain the COM–BOS relationship [29,31]. In this study, increased step width during BW might be a gait adjustment that serves to increase the BOS from internal perturbations caused by BW.

### 4.2. The Effect of Arm Position on Backward Walking

CrossBW had a significantly higher stance phase (%) and DSP but a lower swing phase (%) compared to AbBW. Although our results do not indicate significant differences between CrossBW and NBW, a previous study showed that forward walking with crossed arms led to a significantly higher DSP (%) than independent arm swing during forward walking [32]. Arm swing is an important action in walking. Several studies have reported a decrease in the movement coordination of the upper and lower limbs, which results in gait pattern changes [33], decreased gait velocity [32,34,35], and stride length [36] during FW without arm swing.

Arm swing was restricted during CrossBW, which may have caused gait instability. In contrast, bilateral arm abduction during AbBW can act as a counterbalance, which helps stabilize gait [34] and restore balance when gait is perturbed [37]. Arm abduction was noted to stabilize posture when symmetrical vertical perturbations occurred while standing (sudden free falls) [38]. Moreover, arm muscle activation was initiated as fast as lower limb responses (<100 ms) to stabilize the upright stance following balance perturbations [39]. Biomechanical studies on FW have shown that arm movements are an integral component of human gait that maintain stability and improve gait performance [20,34,35]. AbBW may result in greater activation of the backward gait stabilizer system compared to CrossBW and NBW. We support the hypothesis that lower contralateral upper limb coordination plays an important role in gait locomotion, during either forward or backward walking [20,40,41].

### 4.3. The Effect of Visual Feedback on Backward Walking

The other major finding of this study is that ClosBW demonstrated significantly higher ML COM displacement than NBW. A previous study observed that FW requires visual-vestibular feedback for lateral stability, which is affected by eye closure in young adults [42]. Similarly, a previous study suggested that visual feedback is important for lateral stabilization [43]. According to Dakin and Rosenberg [44], visual information is transformed from an eye-centered reference frame into a gravity-centered reference frame to maintain upright postural stability. Removal of visual information leads to increased postural sway of the center of gravity, resulting in instability when upright.

Object information, walkway distance, obstacles, and the surrounding environment are perceived through the eyes and used to plan and execute anticipatory actions at a higher level of cognitive function before walking. While walking, the dorsal stream uses real-time information on spatial location and objects in the visual field to adjust gait and maintain stability [45]. If errors or unexpected events are detected with visual input, rapid gait correction adjusts the interlimb trajectories in about 120 ms [46]. Thus, the coordination between visual input and muscular reaction (visuomotor) is important for proper movement and safety. NBW was performed with fully operated visuomotor functions, while ClosBW reduced visuomotor function, which contributed to the more significant whole-body sway [47,48]. Typically, the inhibition of sensory input evokes other sensory responses to maintain balance [49,50]. Therefore, the removal of visual information may result in gait instability that stimulates somatosensory functions. However, removing visual cues in ClosBW, which is not a daily activity, may be too challenging to provoke somatosensory assistance. Thus, ML COM during ClosBW is evidently higher.

Despite the significant increase in ML COM due to the removal of visual feedback, there were no changes in VT COM in the present study. Recently, several studies [15,25,28] have shown that hip joint movements and hip muscle activity decrease when walking backward. These changes may cause decreased pelvic rotation and tilt, which are related to decreased walking step lengths. The similarity of the step lengths in both ClosBW and NBW may explain the comparable VT COM displacements, as step lengths affect VT COM but not ML COM. Conversely, the ML COM displacements were increased by shifting the body weight during walking to widen step widths [31]. The biomechanics of COM displacements and joint angles in divergent planes during these specific backward walking conditions may require further analysis.

### 4.4. Implications and Limitations

The present study has demonstrated the remarkable effects of increased speeds, restricted arm swing, and closed eyes on the COM displacements and spatiotemporal parameters of backward gait. Furthermore, BW conditions may require the ability to perform gait adjustments, which are important for assessing balance mobility. Thus, BW may challenge the gait stabilizing system more than FW, improving sensitivity during analysis. However, changes in toe clearance, lower limb joint angle, and the margin of stability during BW have yet to be measured. In addition, the relationship between various BW conditions and an individual’s objective balance assessment should also be identified. Ultimately, we aim to develop a balance mobility assessment tool based on the BW task.

One limitation of this study is that we did not measure joint angles and muscle activity during BW, which could be relevant to identifying fall risk. Furthermore, the participants in this study were young and healthy and are not expected to be the target users of balance assessments. Nonetheless, as we were mainly concerned about the effects of various types of BW on gait parameters, healthy participants allowed us to determine these effects of BW and provide a baseline for future work. The changes in BW due to age, as well as fall history and other impairments, will be investigated in future studies that build on the results of this study.

## 5. Conclusions

We demonstrated that backward walking with increased speed, crossed arms, and closed eyes conditions can induce internal perturbations that require gait adjustment to maintain balance. We argue that BW is a promising tool for developing more sensitive balance mobility assessments.

## Figures and Tables

**Table 1:** Characteristics of the participants.

Characteristics Data	Male (*n* = 11)	Female (*n* = 12)
Age (years)	23.4 ± 1.3	22.8 ± 1.8
Mass (kg)	62.8 ± 6.9	54.8 ± 10.5
Height (m)	1.75 ± 0.1	1.62 ± 0.1
BMI (kg/m^2^)	20.4 ± 2.2	20.9 ± 3.5

The values are determined with descriptive statistics.

**Table 2:** Effects of walking direction and speed.

	Normal Speed		Fast Speed		*p*-Value		
Parameter	NFW	NBW	FFW	FBW	Speed	Direction	Interaction
**Spatiotemporal parameter measures**							
Velocity (m/s)	1.24 ± 0.18	0.99 ± 0.20	1.79 ± 0.26	1.45 ± 0.26	<0.001 ⁑	<0.001 ⁑	0.052
Cadence (steps/min)	113.7 ± 9.8	106.9 ± 15.2	138.9 ± 12.5	138.7 ± 18.5	<0.001 ⁑	0.091	0.023 *
Step length (mm)	651.9 ± 66.0	552.5 ± 68.9	775.4 ± 96.5	631.4 ± 89.6	<0.001 ⁑	<0.001 ⁑	0.002 *
Step width (mm)	171.5 ± 23.9	200.4 ± 35.5	177.8 ± 26.9	217.4 ± 33.5	0.001 *	<0.001 ⁑	0.059
Walk ratio (mm/steps/min)	5.77 ± 6.7	5.26 ± 8.9	5.63 ± 8.9	4.64 ± 9.3	0.009 *	<0.001 ⁑	0.091
**Phases of gait cycle measures**							
Stance phase (s)	0.62 ± 0.06	0.68 ± 0.12	0.48 ± 0.05	0.49 ± 0.7	<0.001 ⁑	0.012 *	0.019 *
Swing phase (s)	0.44 ± 0.04	0.47 ± 0.06	0.40 ± 0.03	0.40 ± 0.05	<0.001 ⁑	0.042 *	0.008 *
DSP phase (s)	0.09 ± 0.02	0.11 ± 0.03	0.04 ± 0.02	0.05 ± 0.02	<0.001 ⁑	0.021 *	0.140
Stance phase (%)	58.6 ± 1.4	59.3 ± 1.8	54.5 ±1.6	54.7 ± 2.2	<0.001 ⁑	0.173	0.392
Swing phase (%)	41.4 ± 1.4	40.7 ± 1.8	45.5 ± 1.6	45.3 ± 2.2	<0.001 ⁑	0.173	0.392
DSP (%)	8.5 ± 1.3	9.0 ± 1.8	4.5 ± 1.4	4.8 ± 2.1	<0.001 ⁑	0.199	0.690
**COM displacement measures**							
ML COM (mm)	34.2 ± 10.1	42.6 ± 16.6	35.0 ± 10.0	44.1 ± 18.2	0.633	0.009 *	0.879
VT COM (mm)	32.1 ± 7.1	39.3 ± 10.8	38.8 ± 13.4	44.1 ±14.1	<0.001 ⁑	0.003 *	0.422

Note: * Significant difference (*p* < 0.05); ⁑ Significant difference (*p* < 0.001). NFW—normal forward walking; NBW—normal backward walking, FFW—fast forward walking, FBW—fast backward walking.

**Table 3:** Effects of arm position on backward walking.

Parameters	Backward Walking in Different Arm Positions				
	NBW	CrossBW	AbBW	F	*p*-Value
**Spatiotemporal parameter measures**					
Velocity (m/s)	0.99 ± 0.2	0.94 ± 0.2	0.99 ± 0.2	0.556	0.576
Cadence (steps/min)	106.9 ± 15.2	105.8 ± 14.7	105.9 ± 16.4	0.034	0.967
Step length (mm)	552.5 ± 68.9	529.5 ± 80.5	564.3 ± 70.7	1.331	0.271
Step width (mm)	200.4 ± 35.5	207.2 ± 40.0	199.2 ± 35.5	0.311	0.733
Walk ratio (mm/steps/min)	5.26 ± 0.9	5.07 ± 0.9	5.43 ± 1.0	0.877	0.421
**Phases of gait cycle measures**					
Stance phase (s)	0.68 ± 0.12	0.69 ±0.12	0.68 ± 0.13	0.103	0.902
Swing phase (s)	0.47 ± 0.06	0.46 ± 0.54	0.48 ± 0.07	0.678	0.511
DSP phase (s)	0.11 ± 0.03	0.11 ± 0.03	0.10 ± 0.04	0.733	0.484
Stance phase (%)	59.3 ± 1.8	60.0 ± 2.1	58.5 ± 2.1	3.382	0.040 *
Swing phase (%)	40.7 ± 1.8	40.0 ± 2.1	41.5 ± 2.1	3.381	0.040 *
DSP (%)	9.0 ± 1.8	9.8 ± 1.8	8.2 ± 2.0	3.974	0.023 *
**COM displacement measures**					
ML COM (mm)	42.6 ± 16.6	48.3 ± 20.0	47.8 ± 16.2	0.484	0.484
VT COM (mm)	39.3 ± 10.8	38.0 ± 11.6	36.8 ± 10.0	0.751	0.751

Note: * Significant difference (*p* < 0.05). NBW—normal backward walking; CrossBW—crossed arms backward walking, AbBW—abducted arms backward walking.

**Table 4:** Effect of visual feedback during backward walking.

	BW in Different Visual Feedback Conditions		
Parameters	NBW	ClosBW	*p*-Value
**Spatiotemporal parameter measures**			
Velocity (m/s)	0.99 ± 0.20	0.96 ± 0.2	0.626
Cadence (steps/min)	106.9 ± 15.2	108.1 ± 14.7	0.782
Step length (mm)	552.5 ± 68.9	527.2 ± 90.1	0.291
Step width (mm)	200.4 ± 35.5	211.3 ± 35.6	0.306
Walk ratio (mm/steps/min)	5.26 ± 8.9	4.93 ± 0.9	0.218
**Phases of gait cycle measures**			
Stance phase (s)	0.68 ± 0.12	0.67 ±0.13	0.814
Swing phase (s)	0.47 ± 0.06	0.46 ± 0.05	0.573
DSP phase (s)	0.11 ± 0.03	0.10 ± 0.04	0.874
Stance phase (%)	59.3 ± 1.8	59.0 ± 2.0	0.573
Swing phase (%)	40.7 ± 1.8	41.0 ± 2.0	0.782
DSP (%)	9.0 ± 1.8	8.8 ± 2.0	0.703
**COM displacement measures**			
ML COM (mm)	42.6 ± 16.6	84.2 ± 25.2	<0.001 ⁑
VT COM (mm)	39.3 ± 10.8	36.6 ± 8.9	0.359

Note: ⁑ Significant difference (*p* < 0.001). NBW—normal backward walking (open eyes); ClosBW—closed eyes backward walking.

## Data Availability

Not Applicable.

## References

[1] World Health Organization Falls. https://www.who.int/news-room/fact-sheets/detail/falls.

[2] Brians L.K., Alexander K.C., Grota P.A., Chen R.W., Dumas V. (1991). The Development of the RISK Tool for Fall Prevention. Rehabil. Nurs. J..

[3] Robinovitch S.N., Feldman F., Yang Y., Leung P., Health F., Sims-Gould J., Robinovitch S., Robinovitch S., Feldman F., Yang Y. (2013). Video Capture of the Circumstances of Falls in Elderly People Residing in Long-Term Care: An Observational Study. Lancet.

[4] Levine D., Richards J., Whittle M. (2012). Whittle’s Gait Analysis.

[5] Steffen T.M., Hacker T.A., Mollinger L. (2002). Age- and Gender-Related Test Performance in Community-Dwelling Elderly People: Six-Minute Walk Test, Berg Balance Scale, Timed Up & Go Test, and Gait Speeds. Phys. Ther..

[6] Panella L., Tinelli C., Buizza A., Lombardi R., Gandolfi R. (2008). Towards Objective Evaluation of Balance in the Elderly: Validity and Reliability of a Measurement Instrument Applied to the Tinetti Test. Int. J. Rehabil. Res..

[7] Pardasaney P.K., Latham N.K., Jette A.M., Wagenaar R.C., Ni P., Slavin M.D., Bean J.F. (2012). Sensitivity to Change and Responsiveness of Four Balance Measures for Community-Dwelling Older Adults. Phys. Ther..

[8] King G.W., Abreu E.L., Cheng A.-L., Chertoff K.K., Brotto L., Kelly P.J., Brotto M. (2016). A Multimodal Assessment of Balance in Elderly and Young Adults. Oncotarget.

[9] Carter V., Jain T., James J., Cornwall M., Aldrich A., de Heer H.D. (2019). The 3-m Backwards Walk and Retrospective Falls: Diagnostic Accuracy of a Novel Clinical Measure. J. Geriatr. Phys. Ther..

[10] Fritz N.E., Worstell A.M., Kloos A.D., Siles A.B., White S.E., Kegelmeyer D.A. (2013). Backward Walking Measures Are Sensitive to Age-Related Changes in Mobility and Balance. Gait Posture.

[11] Taulbee L., Yada T., Graham L., O’Halloran A., Saracino D., Freund J., Vallabhajosula S., Balasubramanian C.K. (2021). Use of Backward Walking Speed to Screen Dynamic Balance and Mobility Deficits in Older Adults Living Independently in the Community. J. Geriatr. Phys. Ther..

[12] Kurz M.J., Wilson T.W., Arpin D.J. (2012). Stride-Time Variability and Sensorimotor Cortical Activation during Walking. Neuroimage.

[13] Chang K.-W., Lin C.-M., Yen C.-W., Yang C.-C., Tanaka T., Guo L.-Y. (2021). The Effect of Walking Backward on a Treadmill on Balance, Speed of Walking and Cardiopulmonary Fitness for Patients with Chronic Stroke: A Pilot Study. Int. J. Environ. Res. Public Health.

[14] Wang J., Yuan W., An R. (2018). Effectiveness of Backward Walking Training on Spatial-Temporal Gait Characteristics: A Systematic Review and Meta-Analysis. Hum. Mov. Sci..

[15] Lee M., Kim J., Son J., Kim Y. (2013). Kinematic and Kinetic Analysis during Forward and Backward Walking. Gait Posture.

[16] Laufer Y. (2005). Effect of Age on Characteristics of Forward and Backward Gait at Preferred and Accelerated Walking Speed. J. Gerontol. Ser. A Biol. Sci. Med. Sci..

[17] Mirelman A., Shema S., Maidan I., Hausdorff J.M., Day B.L., Lord S.R. (2018). Chapter 7—Gait. Handbook of Clinical Neurology.

[18] Weerdesteyn V., Hollands K.L., Hollands M.A., Day B.L., Lord S.R. (2018). Chapter 8—Gait Adaptability. Handbook of Clinical Neurology.

[19] Bruijn S.M., Meijer O.G., van Dieën J.H., Kingma I., Lamoth C.J.C. (2008). Coordination of Leg Swing, Thorax Rotations, and Pelvis Rotations during Gait: The Organisation of Total Body Angular Momentum. Gait Posture.

[20] Bovonsunthonchai S., Hiengkaew V., Vachalathiti R., Vongsirinavarat M., Tretriluxana J. (2012). Effect of Speed on the Upper and Contralateral Lower Limb Coordination during Gait in Individuals with Stroke. Kaohsiung J. Med. Sci..

[21] Ehara Y., Yamamoto S. (2001). Introduction to Body-Dynamics—Analysis of Standing up Movement.

[22] Orendurff M., Segal A., Klute G., Berge J., Rohr E., Kadel N. (2004). The Effect of Walking Speed on Center of Mass Displacement. J. Rehabil. Res. Dev..

[23] Lu H.-L., Kuo M.-Y., Chang C.-F., Lu T.-W., Hong S.-W. (2017). Effects of Gait Speed on the Body’s Center of Mass Motion Relative to the Center of Pressure during over-Ground Walking. Hum. Mov. Sci..

[24] Tesio L., Rota V. (2019). The Motion of Body Center of Mass During Walking: A Review Oriented to Clinical Applications. Front. Neurol..

[25] Grasso R., Bianchi L., Lacquaniti F. (1998). Motor Patterns for Human Gait: Backward Versus Forward Locomotion. J. Neurophysiol..

[26] Laufer Y. (2003). Age- and Gender-Related Changes in the Temporal-Spatial Characteristics of Forwards and Backwards Gaits. Physiother. Res. Int..

[27] Bogen B., Moe-Nilssen R., Ranhoff A.H., Aaslund M.K. (2018). The Walk Ratio: Investigation of Invariance across Walking Conditions and Gender in Community-Dwelling Older People. Gait Posture.

[28] Thorstensson A. (1986). How Is the Normal Locomotor Program Modified to Produce Backward Walking?. Exp. Brain Res..

[29] Rogers M.W., Mille M.-L., Day B.L., Lord S.R. (2018). Chapter 5—Balance Perturbations. Handbook of Clinical Neurology.

[30] Rothwell J. (1994). Control of Human Voluntary Movement.

[31] Rose J., Gamble J.G. (2006). Human Walking.

[32] Koo H.-M., Lee S.-Y. (2016). Gait Analysis on the Condition of Arm Swing in Healthy Young Adults. Phys. Ther. Rehabil. Sci..

[33] Ford M.P., Wagenaar R.C., Newell K.M. (2007). Arm Constraint and Walking in Healthy Adults. Gait Posture.

[34] Punt M., Bruijn S.M., Wittink H., van Dieën J.H. (2015). Effect of Arm Swing Strategy on Local Dynamic Stability of Human Gait. Gait Posture.

[35] Bruijn S.M., Meijer O.G., Beek P.J., van Dieën J.H. (2010). The Effects of Arm Swing on Human Gait Stability. J. Exp. Biol..

[36] Eke-Okoro S.T., Gregoric M., Larsson L.E. (1997). Alterations in Gait Resulting from Deliberate Changes of Arm-Swing Amplitude and Phase. Clin. Biomech..

[37] Meyns P., Molenaers G., Desloovere K., Duysens J. (2014). Interlimb Coordination during Forward Walking Is Largely Preserved in Backward Walking in Children with Cerebral Palsy. Clin. Neurophysiol..

[38] Sanders O.P., Savin D.N., Creath R.A., Rogers M.W. (2015). Protective Balance and Startle Responses to Sudden Freefall in Standing Humans. Neurosci. Lett..

[39] McIlroy W.E., Maki B.E. (1995). Early Activation of Arm Muscles Follows External Perturbation of Upright Stance. Neurosci. Lett..

[40] Udochkina L.A., Vorontsova O.I., Mazin I.G., Goncharova L.A., Akhmineeva A.K. (2018). Kinematic Parameters of Movement at the Shoulder Joint in Normal Gait in Humans. Neurosci. Behav. Physiol..

[41] Ortega J.D., Fehlman L.A., Farley C.T. (2008). Effects of Aging and Arm Swing on the Metabolic Cost of Stability in Human Walking. J. Biomech..

[42] Bauby C.E., Kuo A.D. (2000). Active Control of Lateral Balance in Human Walking. J. Biomech..

[43] Wuehr M., Schniepp R., Pradhan C., Ilmberger J., Strupp M., Brandt T., Jahn K. (2013). Differential Effects of Absent Visual Feedback Control on Gait Variability during Different Locomotion Speeds. Exp. Brain Res..

[44] Dakin C.J., Rosenberg A., Day B.L., Lord S.R. (2018). Chapter 3—Gravity Estimation and Verticality Perception. Handbook of Clinical Neurology.

[45] Goodale M.A. (1996). Visuomotor Modules in the Vertebrate Brain. Can. J. Physiol. Pharmacol..

[46] Reynolds R., Day B. (2006). Visual Guidance of the Human Foot during a Step. J. Physiol..

[47] Forbes P.A., Chen A., Blouin J.-S., Day B.L., Lord S.R. (2018). Chapter 4—Sensorimotor Control of Standing Balance. Handbook of Clinical Neurology.

[48] Nashner L., Berthoz A. (1978). Visual Contribution to Rapid Motor Responses during Postural Control. Brain Res..

[49] Frost R., Skidmore J., Santello M., Artemiadis P. (2015). Sensorimotor Control of Gait: A Novel Approach for the Study of the Interplay of Visual and Proprioceptive Feedback. Front. Hum. Neurosci..

[50] Peterka R.J., Day B.L., Lord S.R. (2018). Chapter 2—Sensory Integration for Human Balance Control. Handbook of Clinical Neurology.

